# Congenital Intralabyrinthine Cholesteatoma

**DOI:** 10.1155/2014/172162

**Published:** 2014-06-26

**Authors:** Sanjay Prasad, Kiran Prasad, Roya Azadarmaki

**Affiliations:** ^1^Metropolitan NeuroEar Group, The Tower Building, 1101 Wootton Parkway, Suite 900, Rockville, MD 20817, USA; ^2^Columbia University, New York City, NY, USA

## Abstract

A patient with a congenital intralabyrinthine cholesteatoma is presented. High-resolution computerized tomographic scans and intraoperative photomicrographs display features of intralabyrinthine extension. We discuss pathogenetic theories for the development of congenital intralabyrinthine cholesteatoma. The distinction of this condition from congenital cholesteatoma with labyrinthine erosion is discussed.

## 1. Introduction

The presence of a white spheroid mass in the anterosuperior mesotympanum seen through an intact tympanic membrane in an otherwise asymptomatic patient, without prior history of otologic surgery, is diagnostic for congenital cholesteatoma. Ossicular erosion can occur and in unusual cases, extension into the mastoid can be seen [1]. Congenital cholesteatoma arising primarily in the mastoid can erode dural plates and lead to dural involvement [2, 3]. Unlike these dural plates, the otic capsule provides a significant barrier for entry into the labyrinth. Labyrinthine erosion is rare, but when present, typically involves erosion of the lateral semicircular canal with exposure of the membranous labyrinth [3, 4]. Intralabyrinthine spread is exceedingly rare.

We describe the first known case of a patient with congenital intralabyrinthine cholesteatoma. High resolution computerized tomographic (HRCT) scans of the temporal bone and intraoperative photomicrographs display features of intralabyrinthine origin and extension. We discuss pathogenetic theories and discuss the distinction of congenital intralabyrinthine cholesteatoma from congenital cholesteatoma with labyrinthine erosion.

## 2. Case Report

A 27-year-old male presented with a long history of right hearing loss. There was no history of tinnitus, disequilibrium, vertigo or facial paresis/paralysis. The past medical history was significant for trauma to the head from a basketball at the age of two. There was no history of fracture or concussion. There was no family history of conductive hearing impairment.

Otoscopic examination revealed normal tympanic membranes with no visible mass in the mesotympanum. The remaining parts of the otolaryngologic examination were normal.

**Figure 1:**
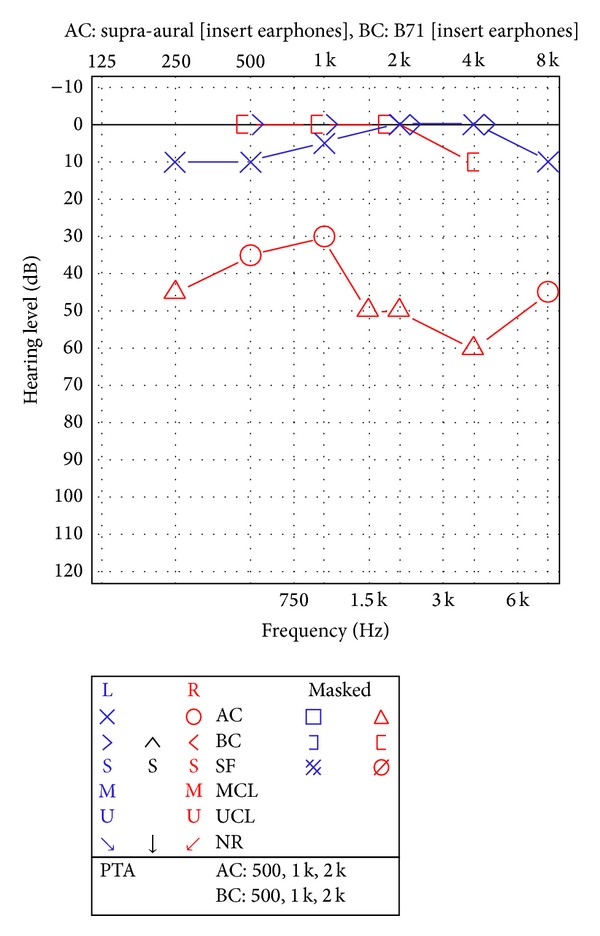
Audiometry revealed a moderate right conductive hearing impairment with excellent speech discrimination scores.

**Figure 2:**
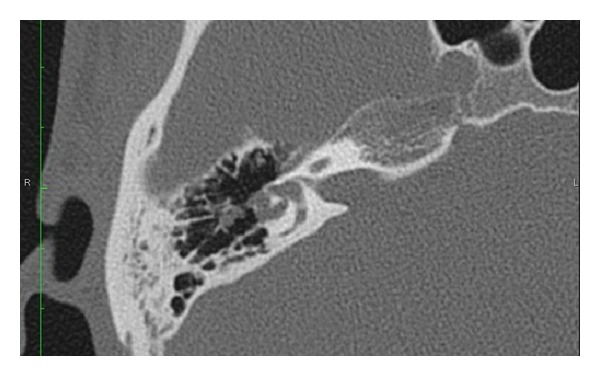
An axial high-resolution computerized tomographic scan shows dilation of the horizontal semicircular canal.

Audiometry revealed a right moderate conductive hearing loss with excellent speech discrimination scores and an absent right stapes reflex to ipsilateral and contralateral stimulation (Figure 1). A HRCT scan of the temporal bone revealed a mass eroding and internally dilating the lateral semicircular canal (Figure 2).

**Figure 3:**
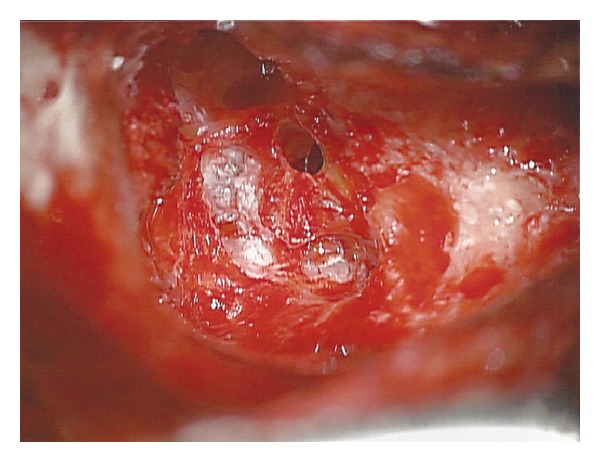
A photomicrograph of the right mastoidectomy defect with the “bony cap” removed shows cholesteatoma within the superior semicircular canal.

**Figure 4:**
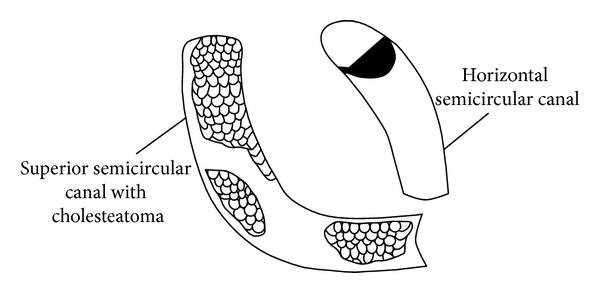
A schematic of Figure 3.

A mastoidectomy operation revealed a “bony cap” over the labyrinth. When the “cap” was removed, cholesteatoma was seen filling the horizontal and superior semicircular canals (Figures 3 and 4). The cholesteatoma was fragile and removed in a piecemeal fashion.

Postoperatively, the patient experienced a temporary vestibulopathy and right anacusis. Pathologic examination revealed cholesteatoma. During a second-look procedure, a formal labyrinthectomy was performed with removal of additional fragments of cholesteatoma. Following the latter procedure, the vestibulopathy nearly resolved.

## 3. Discussion

An astute clinician can make the diagnosis of congenital cholesteatoma. A white mass, in the anterosuperior mesotympanum seen through an intact tympanic membrane, in a patient with no prior history of otologic surgery is diagnostic for this condition. Congenital cholesteatoma can cause ossicular erosion and conductive hearing impairment; however, labyrinthine erosion is rare. Labyrinthine involvement more commonly consists of focal erosion of the horizontal or superior semicircular canal and exposure of the membranous labyrinth. Cholesteatoma matrix can be seen contacting the membranous labyrinth. It is thought that the mechanism of otic capsule erosion is enzymatic destruction or pressure-related remodeling.

Labyrinthine invasion and intralabyrinthine extension, as seen in our case, is exceedingly rare. In our opinion, the tiny volume of middle ear cholesteatoma contiguous with the intralabyrinthine component seen in our case helps confirm an intralabyrinthine site of origin. Spingarn et al. [5] report on a case of “inner ear cholesteatoma”; however, the histology of intralabyrinthine tissue in their case revealed “chronically inflamed granulation and fibroconnective tissue with occasional foreign-body giant cells.” The authors state “one slide contained a small focus of cholesteatoma.” Their findings suggest a case of cholesteatoma causing secondary inflammatory disease of the labyrinth rather than congenital intralabyrinthine cholesteatoma. Jang and Cho [6] report on a patient with congenital cholesteatoma with complete erosion of the pars superior and extension into the internal auditory canal and intracochlear space. The site of origin in this case is difficult to ascertain because of the widespread destruction of the temporal bone.

Many theories have been popularized to explain the genesis of congenital cholesteatoma. Incomplete involution or persistence of the epidermoid formation in the middle ear cleft is the most widely accepted theory [1, 7–9]. Levenson et al. [10] postulate that congenital cholesteatoma results from metaplastic transformation of chronically inflamed middle ear mucosa to keratinizing squamous epithelium. Other theories including abnormal migration of epithelial tissue from the developing external ear canal to the middle ear and seeding of the middle ear cleft by squamous epithelial cells in amniotic fluid have been suggested [1].

These aforementioned theories do not explain the mechanism of entry and extension within the labyrinth. During the third week of embryogenesis, the otic placode, a thickened area of ectoderm adjacent to the rhombencephalon, forms. By the fourth week of development, this otic placode invaginates to form the otocyst, the precursor of the membranous labyrinth. Surrounding neural crest and mesodermal tissue form the otic capsule. Perhaps in congenital intralabyrinthine cholesteatoma, the process of invagination may entrap pluripotential cells that later differentiate into keratinizing squamous epithelium and lead to intralabyrinthine cholesteatoma.

Hearing preservation in congenital cholesteatoma with labyrinthine erosion has been reported [4]. In these cases, it is presumed that the utriculoendolymphatic valve closes and plays a role in protecting the cochlea. Hearing preservation in our case of congenital intralabyrinthine cholesteatoma with such diffuse involvement of the labyrinth was not possible. Future case studies of congenital intralabyrinthine cholesteatoma will be needed to determine whether hearing preservation in patients with less disease extension is possible.

Diagnosis of congenital intralabyrinthine cholesteatoma should be suspected in patients with unilateral conductive hearing loss, as in our case, or anacusis. A mesotympanic mass may not be seen behind an intact tympanic membrane. This case underscores the importance of HRCT imaging in patients with conductive hearing impairment. In congenital intralabyrinthine cholesteatoma, HRCT imaging may reveal dilated intralabyrinthine spaces. The conductive hearing impairment is presumed to be of inner ear origin. Vestibulopathy may or may not be present depending on the degree of central vestibular compensation.

The diagnosis can only be confirmed intraoperatively. Resulting anacusis can be habilitated with use of CROS (contralateral routing of signal) technology or with osseointegrated implants. Postoperative vestibulopathy resolves with central vestibular compensation. In some cases, vestibular rehabilitation may hasten recovery.

## 4. Conclusion

Congenital intralabyrinthine cholesteatoma is exceedingly rare. Suspicion should be aroused in patients with unilateral conductive hearing loss or anacusis. HRCT of the temporal bone can reveal a lesion within the labyrinth with dilation of the intralabyrinthine space. It is the author's opinion that, in most cases, hearing preservation with surgery is not possible.
